# Androgen transition and management of hereditary angioedema long-term prophylaxis in real life: a single-center case series

**DOI:** 10.1186/s13023-024-03251-5

**Published:** 2024-07-09

**Authors:** Cyrille Hoarau, Alireza Maleki, Laurence Bouillet, Isabelle Boccon-Gibod

**Affiliations:** 1 Transversal Allergology and Clinical immunology department, Tours University Hospital, 2 boulevard Tonnellé, Tours, 37000 France; 2 CREAK Competence center of Tours, Tours University Hospital, Tours, 37000 France; 3 ISCHIEMIA, Inserm UMR1327, 10 boulevard Tonnellé, Tours, 37032 France; 4 University Grenoble Alpes, CNRS, UMR 5525, VetAgro Sup, Grenoble INP, Grenoble, 38000 France; 5 French National Reference Center for Angioedema (CREAK), Grenoble University Hospital, Grenoble, 38000 France; 6 Internal Medicine Department, CHU de Grenoble, Grenoble, 38000 France

**Keywords:** Attenuated androgens, Berotralstat, Lanadelumab, C1 inhibitor, Hereditary angioedema

## Abstract

**Background:**

Hereditary angioedema (HAE) is a rare and potentially life-threatening disease that manifests clinically as recurrent episodes of swelling affecting multiple anatomical locations. Long-term prophylaxis (LTP) aims to control the disease by preventing HAE attacks. Previously, treatments such as attenuated androgens have been used for LTP, but they have an unfavorable adverse effect profile. Today, these limitations may be overcome by patients transitioning to newer, targeted therapies including oral berotralstat and subcutaneous lanadelumab. This case series reports the transition process between different prophylactic therapies in a family with HAE in a real-world setting.

**Results:**

Four adult patient cases from the same family who underwent transitions in HAE prophylaxis are presented. Three were female and one male. Two patients who transitioned to berotralstat were initially prescribed attenuated androgens. Two patients were not taking LTP at the time of initiating targeted treatment but had previously been prescribed tranexamic acid. The length of transition varied between the patients, with the longest time taken to stabilize on new therapy being 26 months. All patients received regular follow-up in person or by telephone and all four required an adjustment from their initial treatment plan.

**Conclusions:**

Transitioning between LTP in HAE may help improve control of attacks, avoid unwanted adverse effects, or better cater to individual patient preferences. Newer targeted therapies have been shown to be effective and should be discussed with patients. Shared decision-making is a tool that can aid these discussions. The transition journey between LTP therapies in HAE may not be straightforward and is specific to each patient. Physicians should consider complicating factors such as patient anxieties around changing treatment, adverse effects, preferred routes of administration, and speed of transition. Following patients closely during the transition period helps identify any issues, including difficulties with treatment adherence, and may allow the transition plan to be adapted when necessary.

## Introduction

Hereditary angioedema (HAE) is a rare genetic disorder characterized by episodes of localized, spontaneous soft tissue swelling without urticaria [1, 2]. The swelling can affect multiple locations including the face, upper respiratory tract, extremities and the gastrointestinal (GI) tract [2]. The frequency and length of episodes varies, but episodes may last for days and just over half of those affected experience twelve or more episodes a year [3, 4]. Complications include pain, disability and laryngeal edema potentially leading to life-threatening airway compromise [1]. HAE negatively impacts patients’ work attendance, quality of life, and mental wellbeing; its burden is also felt by patients’ caregivers [5, 6].

HAE is classified by whether the C1-inhibitor (C1-INH) protein is abnormal in quantity or function – Type I and Type II HAE-C1-INH respectively [2]. The etiology of HAE-C1-INH is linked to mutations in the *SERPING1* gene [7]. C1-INH is a key regulator of the kallikrein-kinin pathway (the contact system) [4, 8]. Defects in C1-INH lead to uncontrolled kallikrein activity and therefore excess levels of bradykinin which increases vascular permeability, causing soft tissue swelling [4, 9].

Management of HAE includes treating acute attacks, and preventing attacks in both the short and long term [2, 10]. Acute attacks may be treated with on-demand plasma-derived C1-INH (pdC1-INH) or icatibant [10]. Long-term prophylaxis (LTP) has been shown to reduce the frequency and duration of episodes and therefore prevent life-threatening complications and improve patients’ quality of life [10]. Today, for adults, first-line LTP agents include pdC1-INH, lanadelumab and berotralstat; others include attenuated androgens (AAs) and tranexamic acid [10]. Lanadelumab, administered subcutaneously, and pdC1-INH, available in subcutaneous or intravenous formulations, are effective methods of LTP [11, 12]. However, some patients and caregivers find injectable prophylactic treatments burdensome [5, 13]. Berotralstat is an oral, once-daily plasma kallikrein inhibitor that has been shown to significantly reduce the frequency and duration of angioedema episodes compared with placebo [14, 15].

Some patients with HAE, especially those diagnosed before the availability of newer agents, continue to receive AAs, progestins and tranexamic acid as LTP in light of ease of access, price and patient habituation [16–18]. However, AAs have several limitations related to efficacy, adverse effects (AEs) and contraindications [18]. Adverse effects of AAs include mood disturbances, an increased risk of cardiovascular events, an increased risk of hepatocellular carcinoma, virilization and menstrual irregularities [18]. AAs are contraindicated in pregnancy and in children [10]. In light of these limitations, AAs are no longer recommended as first-line treatment in the 2021 World Allergy Association/ European Academy of Allergy and Clinical Immunology (WAO/EAACI) Guidelines [10].

Transitioning between prophylactic therapies can be a source of concern for patients and healthcare professionals. One such concern is the potential impact on disease control [18].

In patients transitioning from lanadelumab to berotralstat, berotralstat can be initiated concurrently with the final dose of lanadelumab because the latter has a half-life of approximately 2 weeks while berotralstat reaches steady state in 6–12 days [19]. Moreover, there is a lower risk for withdrawal effects associated with stopping lanadelumab than there is with AAs [18, 20].

It has been particularly difficult for patients to transition from AAs owing to rebound attacks, AEs associated with withdrawal, and psychological dependence [18]. While there are no consensus guidelines on how best to stop AAs, several real-world strategies have been described for the transition from AAs to lanadelumab, pdC1-INH and on-demand only therapy [16]. These strategies are based on tapering or overlapping of treatments, or an immediate switch [16, 18]. The latter approach has raised concerns regarding an increase in HAE attacks and other adverse events [18]. It is recommended to avoid abrupt withdrawal of AAs when transitioning to berotralstat [21, 22]. As the main aim of these recommendations is to minimize side effects from AA withdrawal, their validity is also expected when transitioning to other LTP.

Given the variety of therapies and methods to transition between LTP, it is important to consider individual patient preferences in treatment. Treatment choices should therefore be based on principles of shared decision-making (SDM), an adaptive, collaborative model of working between healthcare professionals and patients to find the most appropriate solution for the patient [23]. The importance of SDM in patients with HAE is reflected in the latest WAO/EAACI guidelines for HAE management which state that SDM should be used to determine which of the three first-line LTP to use [10].

Previous case series that have examined the transition between LTP have included pdC1-INH and lanadelumab [16, 24]. Some LTP transitions have been looked at in the context of a clinical trial. APEX-S was an open-label study that aimed to evaluate the long-term safety and effectiveness of berotralstat, concluding that berotralstat was generally well-tolerated and showed durable effectiveness [25]. Within APEX-S separate subgroups of patients who switched from lanadelumab to berotralstat (*n* = 21) or who had prior AA use within 60 days of starting berotralstat (*n* = 39) were analyzed [22, 26]. However there remains a gap in real-world evidence and in our understanding of the transition to berotralstat from other agents.

Here we present a family case series where the similar genetic and environmental factors allow for a comparison of treatment choices and transition protocols. The objective of this case series is to outline the approaches in transitioning between different LTP in HAE and to consider how and why changes to the transition plan were necessary, with the aim of informing future transition guidelines.

## Methods

This retrospective case series describes four patients in the same family identified at Regional University Hospital Centre (CHRU), Tours, France — a partner in the national reference center for angioedema (CREAK) network. Chronological case narratives were described by the center from medical records and experience with the patients. Only descriptive data are provided. Consent was obtained, data were anonymized, and ethics requirements were met.

## Results

### Patient characteristics

**Fig. 1:**
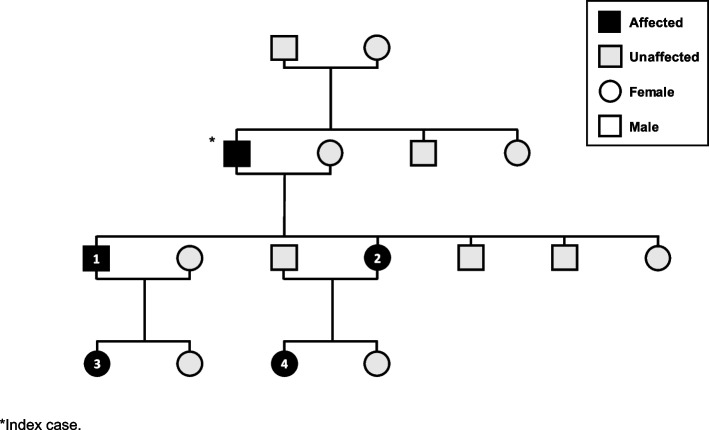
Family tree of included patients

Four members of a family who all had a longstanding diagnosis of HAE (14–45 years), are presented (Table 1). Three patients are female and one male. The relationship between these family members is shown in Fig. 1. A fifth member of the family (the index case) is prescribed AAs by his general practitioner, he has declined any new LTP and as such is not included in this narrative. All four patients were diagnosed with HAE during childhood (between five and 12 years of age) and initially presented with peripheral edema; two also presented with abdominal pain at diagnosis. All four patients had immunology and genetic tests indicative of low C1-INH levels (< 150 mg/L; lab reference range: 210–380 mg/L), confirming type I HAE with the same *SERPING1* exon 4 deletion. All were adults at the time of treatment transition.

**Table 1:** Baseline demographics

Case	Sex	Current age	Mutation	Age at diagnosis	Presenting symptoms at diagnosis	C1-INH level (mg/L)
1	Male	52	*SERPING1* exon 4 deletion	7	Peripheral edema	142
2	Female	50	*SERPING1* exon 4 deletion	12	Peripheral edema and abdominal pain	130
3	Female	21	*SERPING1* exon 4 deletion	5	Hand swelling and abdominal pain	90
4	Female	19	*SERPING1* exon 4 deletion	5	Foot and lower limb edema	50

### Transition process

**Fig. 2:**
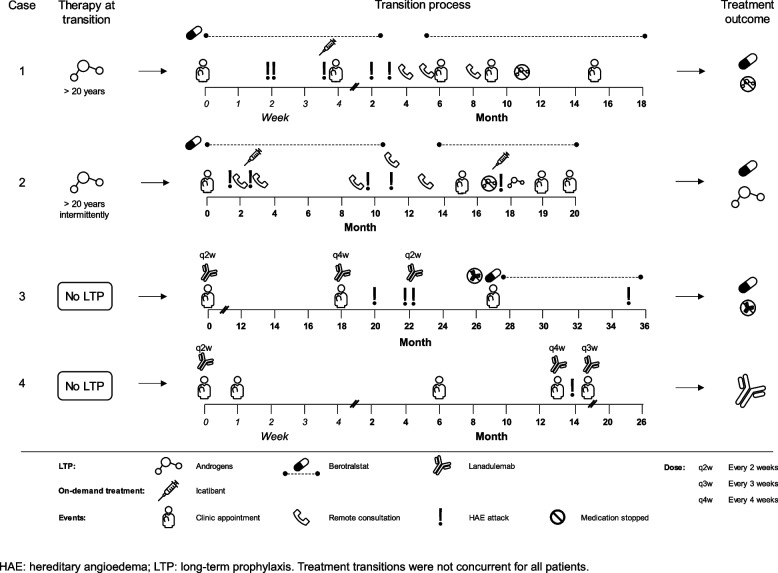
Treatment course

Considering their individual risk–benefit assessment and their personal preferences, all four patients underwent transition to a preferred LTP agent. [10] The timeline for each patient is shown in Fig. 2. For ease of comparison, AA (danazol) doses are expressed as total weekly doses. These were taken as 200 mg single doses by the relevant patients, spread evenly across the week.

#### Patient 1

**Fig. 3:**
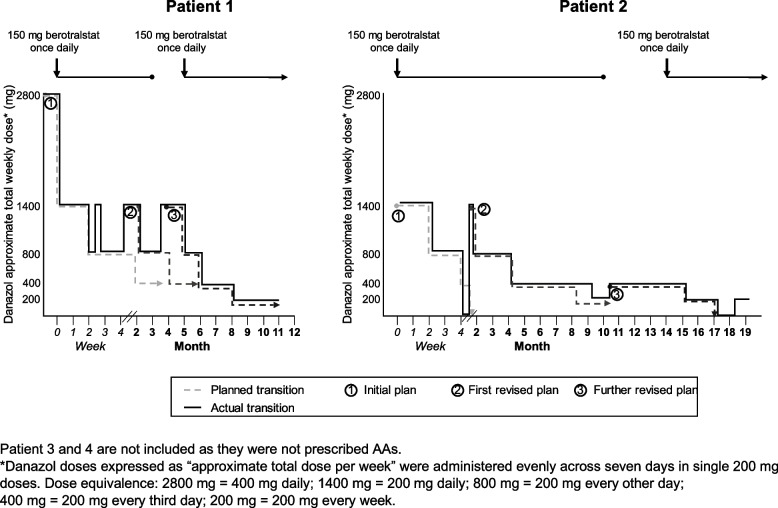
Planned and actual transition process from androgens

This 52-year-old male patient initially presented with peripheral angioedema and was diagnosed with HAE at 7 years old. At the time of transition, he had been treated with AAs (danazol 200 mg daily, total weekly dose 1400 mg) for more than 20 years, with an increase in dose to 400 mg daily (total weekly dose 2800 mg) two years before due to an increase in frequency of attacks. He had tolerated the treatment well and had no attacks in the two years of increased dosing, including no abdominal pains or need for on-demand treatment. The decision to change treatment was made following a discussion with his clinician regarding the risk–benefit balance, including hypertension and oncological risks of androgens [18]. A detailed plan for transition was made (Fig. 3) and a gradual approach was chosen to try to avoid withdrawal effects. Berotralstat 150 mg once daily was started immediately, at the patient’s request, with an androgen transition plan. At a follow-up visit at Month 1, the patient reported deviating from the treatment plan and was taking danazol 200 mg daily (1400 mg total weekly dose; Fig. 3) alongside berotralstat 150 mg daily. He had experienced three attacks since starting berotralstat and reducing his AA dose. The third attack affected his hands and was thought to be triggered by COVID-19 infection.

In a telephone clinic appointment at Month 4, he reported that he was again taking 1400 mg per week. This was owing to the patient’s anxiety at changing treatment and him experiencing two attacks since his last follow-up, one affecting his arms and wrists and another affecting his genitalia, knees and ankles: neither involved the upper airways nor required on-demand treatment (Fig. 2). He stopped taking berotralstat for Month 3 and Month 4. A discussion was had about switching to lanadelumab rather than berotralstat, but the patient perceived injections with lanadelumab as too restrictive. The risks of long-term AA treatment were emphasized again, and a decision was reached with the patient to continue taking a total dose of 1400 mg per week for two months, followed by a further stepwise reduction (Fig. 3). This adapted plan included restarting berotralstat 150 mg daily at Month 5, as co-prophylaxis, and a reduction of danazol to a total weekly dose of 800 mg for one month followed by a further reduction at Month 6 to a 400 mg total weekly dose thereafter with planned telephone consultations to review the dose reductions.

At Month 8 the patient’s HAE was being managed with 200 mg danazol per week. He had no further attacks in the weeks preceding his clinic appointment in Month 9. Two months after this he stopped taking AAs altogether and remained free from any further attacks for 6 months on berotralstat monotherapy.

#### Patient 2

This 50-year-old female patient was diagnosed with HAE at the age of eight after presenting with peripheral edema and abdominal pain. She had taken danazol intermittently for over 20 years, switching to pdC1-INH during her two pregnancies and restarting AAs post-partum. When the decision was made to transition to berotralstat 150 mg daily, she had been taking danazol at a total weekly dose of 1400 mg for approximately seven years. The decision to change was based on an assessment of the risk–benefit balance of AAs in the context of her sex and the availability of newer, targeted first-line LTP for HAE [10]. A plan to reduce danazol gradually over the course of six weeks was made and berotralstat was started at a dose of 150 mg daily at the same time (Fig. 3). During a telephone consultation at Month 2, the patient reported an attack affecting her feet, triggered by exercise. In a further telephone consultation two days later, she was experiencing attacks resulting in abdominal pain. No on-demand treatment had been administered. Danazol was reintroduced at a total weekly dose of 1400 mg and it was planned that she would take this dose for seven days followed by 800 mg in total per week until Month 4. When she was reviewed next, she reported no further attacks. She continued to take 800 mg in total per week for almost two months as planned, then the dose was reduced to 400 mg total per week. She reduced this to 200 mg per week at Month 9, a month later than planned. At Month 10, when taking 200 mg weekly, she had one abdominal attack and decided to increase the dose to 400 mg per week. At the same time, during a discussion with her specialist, a decision was made to stop berotralstat to observe the effect this would have on her HAE attacks. She experienced an attack during the time it was suspended and as such she decided to restart berotralstat at Month 13 with a new AA reduction plan. She decreased her androgen dose from 400 mg weekly to 200 mg weekly at Month 15. She was satisfied with berotralstat, reported no AEs, and had only one moderate HAE attack that was thought to be triggered by a flu-like illness. This process took a total of 17 months compared with the initial plan of 6 weeks. However, she restarted AAs one month after stopping them and continues taking danazol 200 mg weekly with a plan to reduce to 200 mg every 10 days in future.

#### Patient 3

A 21-year-old female with intermittent hand edema and abdominal pain since the age of five was diagnosed soon after presentation owing to her family history. She was previously taking tranexamic acid as LTP, but this was changed to chlormadinone (an androgen receptor antagonist and progestogen) when she was 16 years old due to recurrent attacks requiring on-demand treatment with icatibant [27]. She was unable to tolerate chlormadinone and continued to have flare-ups every two weeks when taking no LTP, which affected her face and caused intense abdominal pain. Due to her HAE attacks, she missed time at school and felt low in mood. The decision was made to start lanadelumab. She experienced headaches and an episode of vomiting in the first month of taking lanadelumab 300 mg every two weeks but otherwise tolerated it well. The attacks stopped with treatment. She was reviewed after 18 months on lanadelumab, and it was decided to increase the time between injections to a four-week interval. However, two months later, she experienced recurrent attacks affecting her face and abdomen. As a result, the dosing interval was reduced again to every two weeks and the attacks stopped. After taking lanadelumab at this frequency for a further five months, the patient asked to switch to berotralstat like her father (patient 1), as the injections negatively affected her quality of life and mood. She started taking 150 mg berotralstat daily one week after her last lanadelumab injection. Other than a mild attack seven months later, that did not require on-demand treatment, she has remained attack-free since the transition.

#### Patient 4

Patient 4 is a 19-year-old female who was diagnosed with HAE when she presented with lower limb edema aged five years old. As a child she was prescribed tranexamic acid as LTP and icatibant for on-demand treatment. Due to her fear of self-administered injections, she reported at age 13 that she was not using icatibant. Owing to multiple attacks, particularly affecting her abdomen, she began lanadelumab 300 mg every two weeks at 15 years of age, with injections being delivered by a nurse. After 13 months, she transitioned to lanadelumab 300 mg every four weeks and experienced an attack, so the frequency of dosing was amended to every three weeks. After being treated with lanadelumab every three weeks for over 2 years, the patient has now learned to self-inject and has stable disease control with no attacks reported at her last two clinic appointments. The patient was satisfied with her LTP and no transition was necessary.

#### Adverse effects during transition

AEs from the new treatments were generally mild and subsided when the patients were established on the new therapy (Table 2). No long-term side effects were reported at the time of data analysis with all patients having taken the new therapy for at least 14 months in total (excluding any interruptions in treatment). Patient 1 reported GI upset in the form of a few episodes of diarrhea and abdominal pain that dissipated without intervention. Patient 4 reported dysmenorrhea, but this was longstanding and thought to be unrelated to both the treatment and the transition. Three patients reported attacks during the transition process, though only one attack in patient 2 required on-demand treatment with icatibant (Fig. 2). Patient 3, who transitioned from lanadelumab to berotralstat, experienced no flare-ups in the transition period.

**Table 2:** Adverse effects

Case	Therapy transitioned from	Therapy transitioned to	AEs attributed to withdrawal of previous treatment	AEs attributed to new treatment	Other issues in transition
1	Danazol 400 mg qd (Total weekly dose 2800 mg)	Berotralstat 150 mg qd	NR	Mild abdominal pain, moderate diarrhea one to three times a day at the beginning of treatment	Anxious to change because settled for many years without attacks
2	Danazol 200 mg qd (Total weekly dose 1400 mg)	Berotralstat 150 mg qd	Nil of note	Nil of note	Nil of note
3	Lanadelumab 300 mg q2w	Berotralstat 150 mg qd	A few episodes of diarrhea and abdominal pain that rapidly resolved	NR	Ongoing dysmenorrhea – unrelated to medication
4	Nil	Lanadelumab 300 mg q3w	N/A	Nil of note	NR

*AEs* Adverse effects, *q2w* Every 2 weeks, *q3w* Every 3 weeks, *qd* Every day, *N/A* Not applicable, *NR* Not recorded

## Discussion

This case series describes four patients from two generations in a family with HAE type I, caused by a single *SERPING1* gene mutation, who all underwent LTP transition to newer therapies. Despite having the same genotype there was variability in patient management, both in relation to the type of treatment given and the transition to new therapy. As such they each received an individualized treatment plan and ultimately three patients were prescribed berotralstat and one lanadelumab. All patients received regular follow-up during the transition in person or by telephone. Those transitioning from AAs to a newer, targeted LTP attended more follow-up visits than the patient transitioning from lanadelumab to berotralstat. Patient 4 who transitioned from no prophylaxis to lanadelumab was stabilized easily on LTP but suffered withdrawal effects after an attempt to space out the lanadelumab doses.

In the transition from AAs, a gradual, rather than abrupt, approach was employed in both patients 1 and 2. Between the two approaches though, patient 2 reduced her AA dose more gradually. In both cases the real reduction was performed more gradually than first intended. A similar need to modify the transition plan has been previously demonstrated in a similar case series where it was necessary to reintroduce AAs and taper more slowly [16]. Both the original plan and the final reduction regimen were slower than the method previously suggested in the literature of reducing to 200 mg danazol every other day for a few weeks then every third day for a few weeks before stopping [18].

Transition approaches to berotralstat with an immediate stop of AAs in type 1 HAE have been described in the past with varying degrees of withdrawal effects and breakthrough attacks [16]. However, current expert opinion suggests avoiding this approach owing to the potential for AEs caused by an abrupt withdrawal of AAs [18, 21]. That said, there is no current consensus or evidence-based guidance on how best to stop AAs under these circumstances, although it is hoped that ongoing research from the Stopping Androgen Treatment in Patients with HAE – Characterization of Reasons and Protocols and Development of Advice for Patients and Physicians (SHAERPA) project will help standardize this process [28]. The individual patient’s wishes should still be taken into account together with any forthcoming guidance.

When transitioning from lanadelumab to berotralstat, it is accepted practice that an immediate switch can be made owing to the overlap in the half-life of lanadelumab and the time taken for berotralstat to achieve steady-state concentration [19]. The case described here reinforces an immediate transition strategy.

This case series demonstrates the utility of the SDM model in three main areas of HAE management: guiding LTP discontinuation; choosing alternative therapy; and managing side effects.

Regarding LTP discontinuation, several patient concerns were apparent in these cases including the occurrence of attacks, concordance with the treatment plan and anxiety around dose reduction. Patients who have had effective disease control with AAs may be reluctant to change treatment and abruptly stopping AAs is sometimes anxiety-provoking [16]. One concern is that if attacks occur during the transition, the patient’s confidence in the new treatment may be compromised. A patient-centered approach was taken in these cases and their involvement in decision making was reflected by the differences in the time taken to transition between LTP therapies. For example, one patient discontinued AAs after an 11-month transition period, whereas another discontinued after 17 months and later restarted AA treatment. A previous case series by Maurer et al*.* has also found heterogeneity in discontinuation strategies for patients prescribed AAs [16]. It was necessary to adapt the initial discontinuation plan in both patients prescribed AAs, one of whom felt anxious, and the reduction of AAs had to be slowed and at times even reversed. The decision to temporarily increase the dose was made by the treating physician in conjunction with the patient at a clinic appointment where this patient had reported further attacks. As such, psychological and physiological aspects surrounding androgen withdrawal should be accounted for in this dynamic process.

Reducing AAs at an acceptable pace to the individual patient and taking an adaptive approach may help with concordance, enable the patient to overcome psychological dependence and increase the chances of them completing transition. Patient non-adherence to prescribed medication is a well-described phenomenon in those with chronic disease, and medication adherence in these patients has been estimated at only 50% [29]. This should be accounted for in the transition process. In this case series patients 1 and 2 were not able to adhere to the initial androgen reduction plan because of attacks. Their adherence to AA treatment prior to this was inferred to be good given that a reduction in AA dose precipitated attacks. Imperfect adherence with berotralstat may have been another explanation for these attacks. Patient 3 received lanadelumab as an injectable therapy administered by the nurse, hence, adherence could be monitored more easily. For this patient, no adherence issues were reported.

In relation to the different LTP options available, there were two notable aspects: preferences for route of administration and the patient’s own experience. Concerns around injections were a major factor impacting treatment decisions – three of the four patients stated that they did not want an injectable treatment. However, patient 4 overcame her childhood fear of self-administered injections when she was older, emphasizing that patient preferences may also change over time. One patient cited their independence and autonomy as a reason why they wished to avoid injectable therapy. The views expressed by the patients in this case series are in line with previous research showing that the majority of patients taking non-oral prophylactic treatment for HAE would prefer an oral alternative and were interested in treatments that were easier to administer [30, 31]. Over 50% of patients surveyed in previous work reported feeling tired of their injections or infusions [13]. It may therefore be beneficial to discuss routes of administration with patients using the principles of SDM as recommended in the WAO/EAACI guidelines [10]. In the patient who changed her mind regarding injectable therapy, a SDM approach, considering decision-making as an iterative process in patients with HAE and accounting for their changing needs and attitudes towards treatment over time, was helpful [23].

The patient’s previous experience is also important in decision-making. For example, patient 3 had seen her father taking berotralstat and wished to take this medication herself. This underscores the importance of eliciting patients’ ideas around treatment in this hereditary disorder where they may have witnessed first-hand the impact of treatment on family members. The experience of patient 2 in noticing an increase in the frequency of attacks when she temporarily stopped berotralstat led to her feeling more confident in its effectiveness. The experience of patients also relates to how long they have lived with HAE. For example, those who have experienced previous attacks including life-threatening attacks may be more anxious regarding a change in therapy, as was seen here. In patients who have had less experience of attacks, it may be more important to discuss the significance of these attacks with them prior to planning LTP transition.

The patient demographics including the sex and age of the patients should be considered in the SDM process as the AE profile of some LTPs and their potential impact on patients’ quality of life may be influenced by this. For example, AAs are linked with virilization and menstrual irregularities in women [18]. This is an important consideration in young women such as patients 3 and 4 (aged 21 and 19 years old, respectively).

Adverse effects should also be considered preemptively in patients already taking AAs. Patients 1 and 2 had not yet experienced any long-term side effects from taking AAs. However, side effects from AAs may develop in the future and therefore a proactive approach was taken to transitioning their LTP to avoid safety issues in the future.

In France, the cost of any HAE treatments is reimbursed [17]. Consequently, the SDM process in this French case series hence focused on clinical and patient factors rather than economic considerations. However, cost may be a factor to be considered as part of SDM when both treatment options are considered equal in other aspects and in other healthcare systems [17, 23]. From the perspective of the costs to the wider healthcare system, the higher costs of newer LTP treatments are reported to be offset by reducing disease burden and therefore direct and indirect costs to the healthcare system [17].

Adverse effects reported by patients in this case series were consistent with data from clinical trials [15, 32, 33]. One patient reported GI upset in the form of diarrhea and abdominal pain, which resolved quickly while berotralstat treatment continued. This is similar to what was seen in the Phase II/III clinical trials, where a constellation of AEs relating to GI disturbance (abdominal pain, vomiting and diarrhea) were among the most commonly reported AEs [15, 32, 33]. Therefore, it may be useful to discuss this with patients initiating berotralstat as part of the SDM process to promote concordance. Patients may have concerns related to other potential AEs from any new LTP and these too should be elicited and addressed.

Novel approaches have been employed in these cases to address some of the challenges in LTP transition. Patients may require multiple follow-up appointments, and this has been aided by remote consultations, in particular telephone consultations. The benefits of consulting in this way include better use of resources and closer collaboration between patients and doctors [34]. Where resources are constrained, remote consultation may allow for more regular follow-up of patients with HAE undergoing a change in LTP. It may also help to identify where there are issues with concordance. This is of particular importance in patients who have been prescribed AAs for a long time and who require close monitoring during the discontinuation period.

Involving an advanced practice nurse (APN) is another innovative approach to managing LTP transition in HAE. The APN is an advanced nurse practitioner role in France and there was a new APN in the unit where this family were treated. The role of the APN is to bridge the gap between the traditional doctor and nurse roles [35]. They have a Master’s level degree in Nursing Science and can adjust or continue prescriptions, which may be useful where dose titration is needed for AAs. They can also monitor concordance with treatment and any AEs in line with WAO/EAACI guideline recommendations [10].

This case series has limitations. By nature of its design, it describes a select group of patients from a single center. Therefore, generalization of the findings to the wider HAE population should be taken with care. Moreover, the retrospective design of this case series needs to be considered.

## Conclusions

In summary, transitioning between LTP in HAE may be required to gain better control of HAE attacks, avoid unwanted AEs or better suit patient preferences. In particular, transitioning away from first generation LTP with AAs is an important step for each HAE patient to minimize side effects from long-term androgen exposure. There are now newer targeted LTP options available, and these should be actively discussed with HAE patients to enable them to make an informed treatment decision together with their treating physician. SDM discussions should take into account patient anxieties around stopping previous treatment and AEs, preferred routes of administration, and speed of transition. Patients should be followed closely during the transition process; there may be a role for an APN to adapt the plan to avoid break-through attacks, optimize observance, reassure the patient, and monitor safety signals.

## Data Availability

The raw patient data that support this study are not openly available due to reasons of sensitivity as they consist of the actual patient files securely stored at CHRU Tours. Extracts of relevant parts of the anonymized data are available from the corresponding author upon reasonable request.

## Abbreviations

AA: Danazol
AAs: Attenuated androgens
APN: Advanced practice nurse
C1-INH: C1-inhibitor
CREAK: French national reference center for angioedema
EAACI: European Academy of Allergy and Clinical Immunology
GI: Gastrointestinal
HAE: Hereditary angioedema
LTP: Long-term prophylaxis
SDM: Shared decision-making
SHAERPA: Stopping Androgen Treatment in Patients with HAE – Characterization of Reasons and Protocols and Development of Advice for Patients and Physicians
WAO: World Allergy Association
